# Chitosan-loaded copper oxide nanocomposite as a promising antiviral alleviates Zucchini yellow mosaic virus infection in squash plants

**DOI:** 10.1186/s12870-026-09268-1

**Published:** 2026-06-23

**Authors:** Eman A. Ahmed, Ahmed Shaaban, Khaulood A. Hemida, Mostafa M. Rady, Ahmed I. Ali, Amro A. Farrag

**Affiliations:** 1 Virus and Phytoplasma Research Department, Plant Pathology Research Institute, Agricultural Research Center, Giza, 12619 Egypt; 2 Agronomy Department, Faculty of Agriculture, Fayoum University, Fayoum, 63514 Egypt; 3 Botany Department, Faculty of Science, Fayoum University, Fayoum, 63514 Egypt; 4 Botany Department, Faculty of Agriculture, Fayoum University, Fayoum, 63514 Egypt; 5 Self-Pollinating Vegetable Crops Research Department, Horticultural Research Institute, Agricultural Research Center, Giza, 12619 Egypt

**Keywords:** ZYMV, Polymerase chain reaction, Sequences, *Cucurbita pepo* L., Plant defense responses, Chitosan, Copper oxide

## Abstract

**Background:**

Chitosan has been reported to induce host defense responses against plant viruses, while copper oxide nanoparticles can directly inhibit viral infection. However, the combined effect of chitosan-loaded copper oxide (CS-CuO) nanocomposites, particularly across different nanosizes, remains unexplored against Zucchini yellow mosaic virus (ZYMV). Therefore, this study investigates the antiviral potential of CS-CuO nanocomposites of varying mean particle sizes (70.33, 30.20, and 17.18 nm) to determine whether size-dependent enhancement of induced resistance occurs in squash plants.

**Methods:**

Three CS-CuO nanocomposites with mean particle sizes of 70.33 nm (CS-CuO_70_), 30.20 nm (CS-CuO_30_), and 17.18 nm (CS-CuO_17_) were chemically synthesized and characterized using transmission electron microscopy and dynamic light scattering (zeta potential analysis). Naturally infected squash samples were collected from open fields in the Beni‑Suef Governorate, Egypt (during the 2025 growing season). ZYMV was molecularly detected by RT‑PCR targeting the P1 gene (primers ZY140F/ZY1084R; annealing 64 °C; 35 cycles), yielding a ~ 944 bp amplicon. The amplicon was sequenced, subjected to phylogenetic analysis, and the obtained sequence was deposited in GenBank under accession number PX572932. Under greenhouse conditions, ZYMV‑inoculated squash plants were foliar‑treated 48 h post‑inoculation with each nanocomposite at 50, 100, and 250 mg/L. Untreated healthy and infected plants served as controls, and all treatments were arranged in a completely randomized block design with five biological replicates per treatment.

**Results:**

ZYMV infection markedly increased oxidative damage, evidenced by elevated malondialdehyde levels, high disease incidence, and severe symptom development. Additionally, viral stress caused a modest increase in osmoprotectants as well as enzymatic (e.g., APX, SOD, CAT, PPO, and GR) and non-enzymatic antioxidants, but severely suppressed photosynthetic pigments and growth parameters. Foliar application of all CS-CuO nanocomposites significantly (*p* ≤ 0.05) mitigated ZYMV-induced damage by reducing lipid peroxidation, enhancing photosynthetic pigments, and further strengthening antioxidant capacity. These coordinated responses collectively reduced disease incidence and severity while improving plant growth. All reported treatment effects were statistically significant (*p* ≤ 0.05) according to two-way analysis of variance. Among treatments, CS-CuO_17_ at 250 mg/L was the most effective, reducing disease incidence and severity by 89.9% and 91.8%, respectively, and markedly enhancing leaf number, shoot and root length, and plant dry weight relative to the untreated infected controls.

**Conclusions:**

Overall, CS–CuO nanocomposites, particularly the smallest formulation, effectively enhanced squash growth and activated defense responses against ZYMV. These findings highlight CS-CuO nanocomposites as promising, eco-friendly antiviral agents and a novel approach for the sustainable management of ZYMV in cucurbit production systems.

**Supplementary Information:**

The online version contains supplementary material available at 10.1186/s12870-026-09268-1.

## Introduction

Cucurbitaceae is a taxonomically diverse plant family widely cultivated under different environmental conditions worldwide. Taxonomically, the Cucurbitaceae family comprises approximately 825 species and 118 genera of plants, collectively referred to as cucurbits [1]. Summer zucchini, also known as squash (*Cucurbita pepo* L.), is a member of this family. It is a commercial, short-season vegetable crop extensively cultivated in arid and semi-arid regions worldwide, including Egypt. China, India, Russia, Ukraine, the United States, Egypt, Mexico, Malawi, Italy, and Spain are the top ten global producers of summer *C. pepo* L. [2]. The edible parts (e.g., fruit and seeds) of *Cucurbita pepo* are rich in fatty acids, dietary proteins, structural carbohydrates (including pectin, non-pectin polysaccharides, and dietary fibers), and vitamins (A, B₁, B₂, C, E, and K). They also contain carotenoids (α- and *β*-carotenes) [3], flavonoids, and essential minerals such as potassium, calcium, magnesium (Mg), iron, manganese, zinc, and selenium [4, 5]. These bioactive constituents have been associated in experimental and nutritional studies with antioxidant, antihyperglycemic, hepatoprotective, and cardiometabolic regulatory activities, largely attributed to their rich flavonoid, carotenoid, tocopherol, and cucurbitacin content [6–8]. Cucurbits are susceptible to approximately 90 different viruses worldwide [9], the majority of which are transmitted by whiteflies and aphids [10, 11]. These pathogens belong to diverse viral families, such as *Geminiviridae* (especially the genus *Begomovirus*), *Potyviridae*, *Bromoviridae*, and *Luteoviridae*, and are reported to cause significant economic losses in global cucurbit production [12]. Prominent examples include the watermelon mosaic virus (WMV), cucumber mosaic virus (CMV), zucchini yellow mosaic virus (ZYMV), and cucurbit aphid-borne yellows virus, all of which are widely distributed across most Mediterranean countries and severely impact yield [12]. Furthermore, three specific potyviruses, papaya ringspot virus (PRSV), watermelon mosaic virus-2, and ZYMV, are recognized as the most prevalent viral pathogens infecting cucurbits [13].

Plant viruses are among the most destructive pathogens affecting agricultural productivity worldwide, causing substantial reductions in crop yield and quality. The resulting economic losses vary depending on the host species, virus strain, environmental conditions, and epidemic intensity, ultimately threatening global food security [14–17]. ZYMV is one of the major economically important viruses attacking cucurbit crops worldwide, causing up to 94% yield loss [18]. It exhibits severe symptoms on squash plants including systemic vein-banding, yellowing, blistering, severe mosaic, leaf deformation, and stunting, as well as twisted and deformed fruits, ultimately leading to drastic yield reductions [18, 19]. First isolated in northern Italy [20], the virus rapidly spread globally due to efficient non-persistent transmission by diverse aphid species and the extensive international trade of cucurbit crops. In Egypt, ZYMV has been isolated from naturally infected squash plants [17, 21–23]. The virus is transmitted from plant to plant by aphids in a non-persistent manner [24]. Approximately 60 aphid species have been reported to transmit ZYMV, with *Myzus persicae* and *Aphis gossypii* exhibiting the highest transmission efficiencies (41% and 35%, respectively) [24, 25]. ZYMV possesses a positive-sense, single-stranded RNA genome of approximately 9.5 kb encapsidated in flexuous, filament-shaped particles [26]. RT-PCR has been widely utilized for the accurate detection of ZYMV [21, 27] due to its high sensitivity, specificity, and rapid diagnostic capability compared with many conventional virus detection methods [23]. The ZYMV genome encodes a polyprotein that is processed into several mature proteins. One of these proteins, encoded by the protease (P1) gene, represents a highly informative genomic region for distinguishing potyviruses [23, 28].

Managing ZYMV and other plant viruses is highly challenging due to the absence of curative antivirals and the rapid, non-persistent nature of insect-mediated transmission [24, 26]. Consequently, growers must rely heavily on pesticide applications to suppress vector populations, despite the inherent limitations of this approach [16]. The excessive use of pesticides exerts severe detrimental impacts on the environment and the ecosystem. Therefore, eco-friendly solutions and new strategic technologies are needed for controlling these viral infections [23, 29].

Nanotechnology has emerged as a promising strategy for the control of agricultural pests and phytopathogens [30]. It aims to develop antipathogenic compounds through the conversion of non-metallic, metallic, and bio-compounds into their respective nanoforms [31, 32]. Nanoforms exhibit superior antiviral efficacy compared to their bulk counterparts due to unique physicochemical properties, including higher surface-to-volume ratios, enhanced reactivity, and improved cellular bioavailability [29, 31]. These distinctive properties enable nanomaterials to interact more efficiently with pathogens and host tissues, thereby inhibiting the growth and spread of infectious agents. Various nanoparticles (NPs) have demonstrated excellent antiviral properties against various plant viruses. These nanomaterials activate plant defense mechanisms and reduce virus accumulation in plant cells. Chitosan (CS) is a natural biopolymer comprising β-(1 → 4)-linked D-glucosamine and N-acetyl-D-glucosamine units, produced via the alkaline deacetylation of crustacean chitin [33]. It is characterized by biocompatibility, non-toxicity, biodegradability, environmental safety, and antimicrobial activity. Furthermore, recent reports indicate that CS alone can effectively induce plant defense responses against various pathogens, including viruses [34], highlighting its potential to be developed as a promising elicitor molecule against viral infections and other plant pathogens [29, 31].

Moreover, copper oxide nanoparticles (CuONPs) have various agricultural applications including promoting plant growth, plant protection, and exerting antifungal and antibacterial activities [32, 35, 36]. Numerous studies have investigated the antimicrobial activity of copper nanoparticles; however, relatively few have addressed their antiviral potential [29, 36, 37]. Nanocomposites of CS with metal oxides represent a novel class of hybrid materials that enhance antimicrobial efficacy and significantly upgrade functional qualities, such as electrical and biological conductivities [38–40]. The excellent synergistic action observed in the CS-CuO nanocomposite may be attributed to the role of CS as a stabilizing and capping matrix, wherein copper ions are complexed by the hydroxyl (–OH) and amino (–NH₂) groups of the chitosan chain, ensuring structural stability and minimizing particle growth [40, 41]. Chitosan also serves as an effective stabilizer due to its metal-chelating capacity, which facilitates the precise synthesis of structured nanoparticles [41, 42]. Regarding plant viral pathogens like ZYMV, a nanomaterial’s antiviral efficacy and cellular uptake depend heavily on its size, while its shape dictates interactions with specific viral or host cell targets [43]. Generally, smaller nanomaterials exhibit superior antiviral activity against plant viruses due to their larger surface areas; however, the underlying mechanisms of these size-dependent interactions within cucurbit-virus pathosystems have not yet been fully explored [44]. As established in the literature, chitosan possesses a high cationic charge capable of electrostatically interacting with viral components while simultaneously serving as a potent elicitor of plant systemic defense pathways [33, 34]. Concurrently, copper is a vital enzymatic cofactor in plant antioxidant systems, and CuONPs have demonstrated an inherent capability to disrupt viral structural integrity [29, 37]. Drawing upon these documented, distinct mechanisms as preliminary justification, this study aims to evaluate the potential activity of the CS‑CuO nanocomposite at three different nanosizes to manage ZYMV in *C. pepo* plants, based on its effects on disease incidence and severity, photosynthetic pigments, osmoprotectants, enzymatic and non-enzymatic antioxidants, and growth indices. Consequently, this research logically hypothesized that the synergistic combination of the CS-CuO nanocomposite at three different nanosizes could directly inactivate viral particles while simultaneously triggering systemic resistance and tolerance responses in ZYMV-inoculated *C. pepo* plants.

## Materials and methods

### Virus isolation and propagation

Plant samples were collected with official authorization from the Plant Pathology Research Institute, Agricultural Research Center, Egypt, in compliance with national regulations governing plant disease research and monitoring. During the summer 2025 growing season, a total of 35 symptomatic leaf samples were randomly collected from open-field *C. pepo* L. plantings located in five geographically separated commercial fields across different locations in the Beni Suef Governorate, Egypt. All sampled plants belonged to the highly susceptible local cv. “Eskandarani” and exhibited characteristic symptoms associated with ZYMV infection, including mosaic, blistering, leaf deformation, and stunting. To exclude potential mixed viral infections, all collected samples were initially screened serologically using the double antibody sandwich enzyme-linked immunosorbent assay (DAS-ELISA) technique. Specific ELISA kits against ZYMV, WMV, CMV, PRSV, and tobacco mosaic virus (TMV) were used, following the procedure described by Clark and Adams [45]. DAS-ELISA was conducted according to the protocol provided by the manufacturer using a commercial ELISA kit obtained from LOEWE Biochemica GmbH (D-82054 Sauerlach, Mühlweg 2a, Germany). Only samples testing positive solely for ZYMV were subsequently subjected to molecular confirmation using RT-PCR with ZYMV-specific primers targeting the highly variable P1 protease-coding region, which generated the expected amplicon size of approximately 944 bp. RT-PCR-positive samples were then used for virus isolation on *Chenopodium amaranticolor* through the single local lesion technique. The purified isolate was mechanically propagated on healthy *C. pepo* cv. “Eskandarani” seedlings under greenhouse conditions to maintain a stable viral source. Inoculated plants were monitored weekly for symptom development (i.e., mosaic intensity, blistering, deformation, and stunting), and successful infection with ZYMV was further confirmed by RT-PCR analysis.

### Molecular identification of the isolated virus

#### Extraction of total RNA and RT-PCR assay

Total RNA was extracted from healthy and ZYMV-infected squash samples using the Geneaid RNA extraction Kit (Geneaid-Taiwan), according to the manufacturer’s protocol. The extracted RNA was isolated and resuspended in 40 μl of RNase-free water. Prior to downstream applications, the concentration (ng/μl) and purity of the isolated RNA were spectrophotometrically assessed using a NanoDrop™ One Spectrophotometer (Thermo Fisher Scientific™ Inc., DE, USA). RNA quality was verified by ensuring that the A_260_/A_280_ absorbance ratios were approximately 2.0, and RNA integrity was confirmed via visual inspection of ribosomal RNA bands on a 1% agarose gel. RT-PCR assay was performed to detect the P1 coding region using the P1 forward primer ZY140F (5’ ATG GCC TCC ATT ATG ATT GGTTC) and the reverse P1-primer ZY1084R (5’- TCC GGT TGC GAC GAA TAG TG-ˋ3) as designed by Maghamnia et al. [28], who previously validated their high specificity for the ZYMV P1 gene and confirmed the absence of cross-reactivity with other closely related potyviruses. RT-PCR was performed using the Verso™ 1-Step RT-PCR ReddyMix™ Kit (Thermo Scientific) in a 25 μl total volume containing 12.5 μl of one-step PCR master mix, 5 μl of nuclease-free water, 3 μl of total RNA, 1.5 μl (10 μM) of specific forward and reverse primers, 0.25 μl Verso enzyme, and 1.25 μl of RT-Enhancer. Complementary DNA (cDNA) synthesis was carried out with 15 min of incubation at 50 °C, followed by an initial denaturation at 95 °C for 3 min, 35 cycles of 94 °C at 30 s, 64 °C for 30 s, and 72 °C for 1 min and a final extension at 72 °C for 5 min. The RNA extraction and subsequent RT-PCR assays were performed in triplicate biological replicates for each sample group. The PCR products, alongside positive controls (previously confirmed ZYMV-infected samples), negative controls (nuclease-free water), and healthy plant samples, were analyzed by electrophoresis for 45 min at 100 V in 1% agarose gel prepared in TAE buffer (1x), stained with EZview (Biomatric, USA), and visualized using a UV transilluminator. The size of the amplified products was estimated using a 100 bp DNA Ladder (Biomatik, USA).

#### Nucleotide sequence and phylogenetic analysis

The ZYMV’s PCR products were purified using the Geneaid Gel and PCR Clean-Up System (Geneaid, Taiwan), and subsequent nucleotide sequencing of the P1 gene was performed by Macrogen Inc. (South Korea). The P1 coding region was specifically targeted for sequencing rather than the full genome because it is recognized as a highly variable and reliable molecular marker that provides sufficient resolution for accurately distinguishing potyvirus isolates. The partial nucleotide sequence of the ZYMV-P1 encoding region, yielding a sequence length of 944 bp, was deposited in the NCBI GenBank database under the accession number (PX572932). This sequence was used in phylogenetic analysis and compared with other ZYMV/P1 genes available in the GenBank database. These reference sequences were selected to represent diverse geographical origins and cucurbit host species, thereby providing a broader epidemiological and evolutionary framework for comparison with the local isolate. Due to the limited availability of independently deposited P1 gene sequences in the NCBI GenBank database, the selected reference isolates included both partial and full-length genome submissions; however, only the homologous P1 coding regions were extracted and aligned to ensure a standardized and accurate comparison. Multiple sequence alignments and phylogenetic tree construction were performed using DNAMAN software version 7.

#### Synthesis and characterization of chitosan-loaded copper oxide (CS-CuO) nanocomposite

Chitosan nanoparticles (CSNPs) were prepared based on the ionic gelation of CS with sodium tripolyphosphate (STPP) anions [46]. Chitosan was stirred in 1% (*v*/*v*) of acetic acid at 400 rpm in a magnetic stirrer for 12 h, and then filtered through a polyvinylidene difluoride (PVDF) syringe filter with a 0.22 μm pore size. At the same time, STPP was prepared at a 0.25% (*w*/*v*) concentration in sterile double-distilled water (DdW) and then filtered using a PVDF syringe filter drop by drop. Using a magnetic stirrer rotating at 800 rpm, CS and STPP of equal volume were cross-linked followed by a 10 min centrifugation at 12,000 rpm. The formulation obtained was resuspended in sterile DdW and ultrasonified at a 28% pulse ratio for 100 s at 4 °C to produce pellets. The CSNPs were purified and evenly dispersed through centrifugation and ultrasonication, a process performed three times. The CuONPs were synthesized via the chemical precipitation standard method described by Pandey et al. [47]. Briefly, this process involved the dropwise addition of an alkaline precipitating agent [e.g., NaOH] to a Cu precursor solution [e.g., CuCl₂·2H₂O or Cu(CH₃COO)₂·H₂O] under continuous magnetic stirring and controlled heating until a stable black precipitate formed. The resulting precipitate was subsequently centrifuged, washed repeatedly with distilled water and ethanol to remove impurities, and oven-dried. The synthesis process was optimized under different conditions (e.g., modulating the precursor concentrations, pH, and reaction temperatures), producing three different sizes, i.e., 5 ± 1.8, 20 ± 2.4, and 50 ± 3.4 nm of CuONPs. This was the second step in the synthesis process following a standard procedure described in previous work [48].

**Fig. 1:**
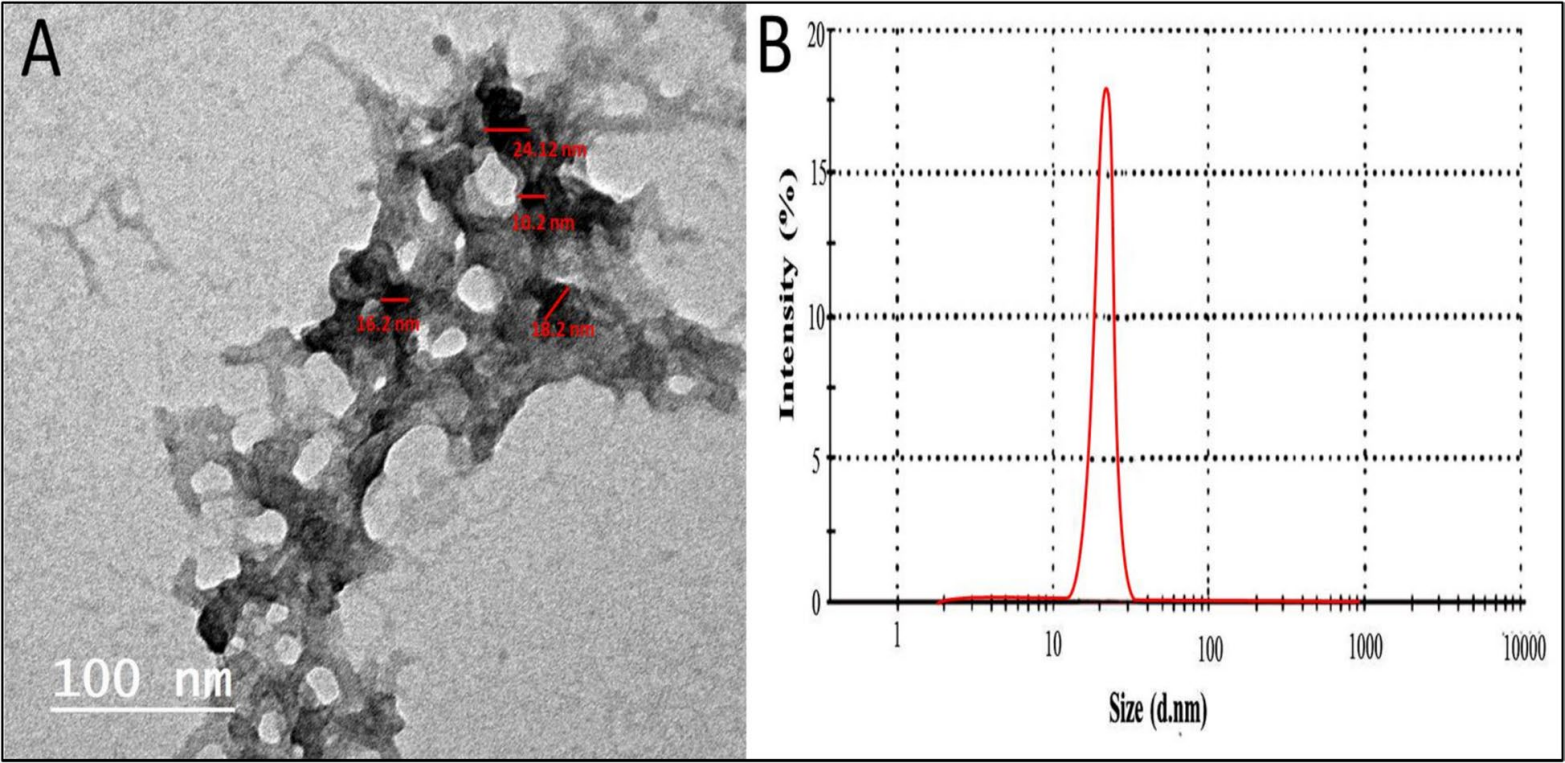
Characterization of the produced CS-CuO nanocomposite (**A**) Transmission electron microscope images of CS-CuO nanocomposite, **B** Dynamic light scattering analysis of the produced CS-CuO nanocomposite

The CS-CuO nanocomposite was prepared by gently mixing 40 mL of the prepared CSNP solution with a 0.5 g of the prepared CuONPs at each nano size (i.e., 5 ± 1.8, 20 ± 2.4, and 50 ± 3.4 nm) and dispered into a homogeneous suspension after sonication for 60 min. The obtained product was then constantly washed with DdW, centrifuged at 8000 rpm for 30 min, dried in a vacuum oven, and stored at 4 °C for future analysis. Characterization of the synthesized CS-CuO nanocomposite was carried out using a transmission electron microscope (TEM, Tecnai 10, Philips, Amsterdam, Netherlands). For this purpose, samples were prepared by diluting 1 mg of CS-CuO nanocomposite separately in 1 mL of DdW. This entire 1 mL suspension was then sonicated for 1 h to ensure complete and homogeneous dispersion. Following sonication, a single drop of the dispersed solution was carefully placed onto carbon coated copper TEM grids that had been previously coated and allowed to dry for 3 h at room temperature, while the extra solution was removed using blotting paper. The size distribution and zeta potential were analyized (Fig. 1A, B) by dynamic light scattering (DLS) with a Zetasizer Ultra (Malvern Panalytical Ltd., Malvern, UK).

#### Greenhouse experimental design and treatments

This experiment was carried out during the 2025 growing season under insect-proof greenhouse conditions at the Fayoum Regional Research Station, Agricultural Research Center (ARC), Egypt. Healthy *Cucurbita pepo* L. cv. Eskandarani seeds were obtained from ARC, Egypt, and were sown in plastic pots (22 cm upper diameter × 35 cm depth) under controlled environmental conditions of 28/16 °C (day/night) with a 70% relative humidity. Ten-day-old squash seedlings were mechanically inoculated with ZYMV-infected plant sap using 0.1 M sodium phosphate buffer (pH 7.2) at a tissue weight: buffer volume ratio of 1:2 as described by Farrag et al. [49]. Eleven treatments were arranged in a randomized complete block design with five independent biological replicates per treatment, where each replicate consisted of one pot containing three squash plants (*n =* 15). The first treatment consisted of uninoculated squash plants that were without a viral inoculation and were foliar sprayed with a sterile nutrient (healthy control; HC). The second treatment consisted of squash plants mechanically inoculated with the ZYMV and then foliar-sprayed with a sterile nutrient (infected control; IC). To specifically evaluate the antiviral efficacy of the complete functional nanocomposite as a final formulated product, all subsequent treatments were compared directly against these standard healthy (HC) and infected (IC) controls, without the inclusion of a standalone CS control. Squash plants assigned to treatments 3–11 (i.e., nine treatments) were foliar-sprayed 48 h after ZYMV inoculation. Each of these nine treatments received one of three different CS‑CuO nanocomposites (CS‑CuO_17_, CS‑CuO_30_, and CS‑CuO_70_), containing CuONPs with mean sizes of 5 ± 1.8 nm, 20 ± 2.4 nm, and 50 ± 3.4 nm, respectively, as detailed in Table 1. Each nanocomposite was applied at three concentrations (50, 100, and 250 mg/L), strategically selected based on previously established effective and non-phytotoxic ranges reported in the literature for CuONP‑based antiviral applications.

**Table 1:** Description of treatments and their concentrations applied on zucchini yellow mosaic virus (ZYMV)-inoculated squash plants in the present study

Treatment	CuONPs size (nm) in nanocomposite	Applied concentration (mg L^−1^)
HC	–-	Untreated
IC	–-	Untreated
CS-CuO_70_ nanocomposite	50	50
		100
		250
CS-CuO_30_ nanocomposite	20	50
		100
		250
CS-CuO_17_ nanocomposite	5	50
		100
		250

*HC* Healthy control (i.e., healthy plants sprayed with double-distilled water only), *IC* Infected control (i.e., untreated zucchini yellow mosaic virus-inoculated squash plants), and CS-CuO nanocomposite = nanocomposite of chitosan-loaded copper oxide (both in nano-form), *CuONPs* Copper oxide nanoparticles

### Observations, measurements, and data collection

#### Disease incidence and severity of ZYMV

The disease incidence and severity of ZYMV on all treated and untreated squash plants were determined three weeks post-inoculation based on the visual symptoms developed on diseased plants. The disease incidence percentage (DI%) was estimated using the following formula:

$$\mathrm{Disease}\;\mathrm{incidence}\;\left(\mathrm{DI}\%\right)=\frac{\mathrm{Number}\;\mathrm{of}\;\mathrm{infected}\;\mathrm{plants}}{\mathrm{Total}\;\mathrm{number}\;\mathrm{of}\;\mathrm{assessed}\;\mathrm{plants}}\times100$$

**Fig. 2:**
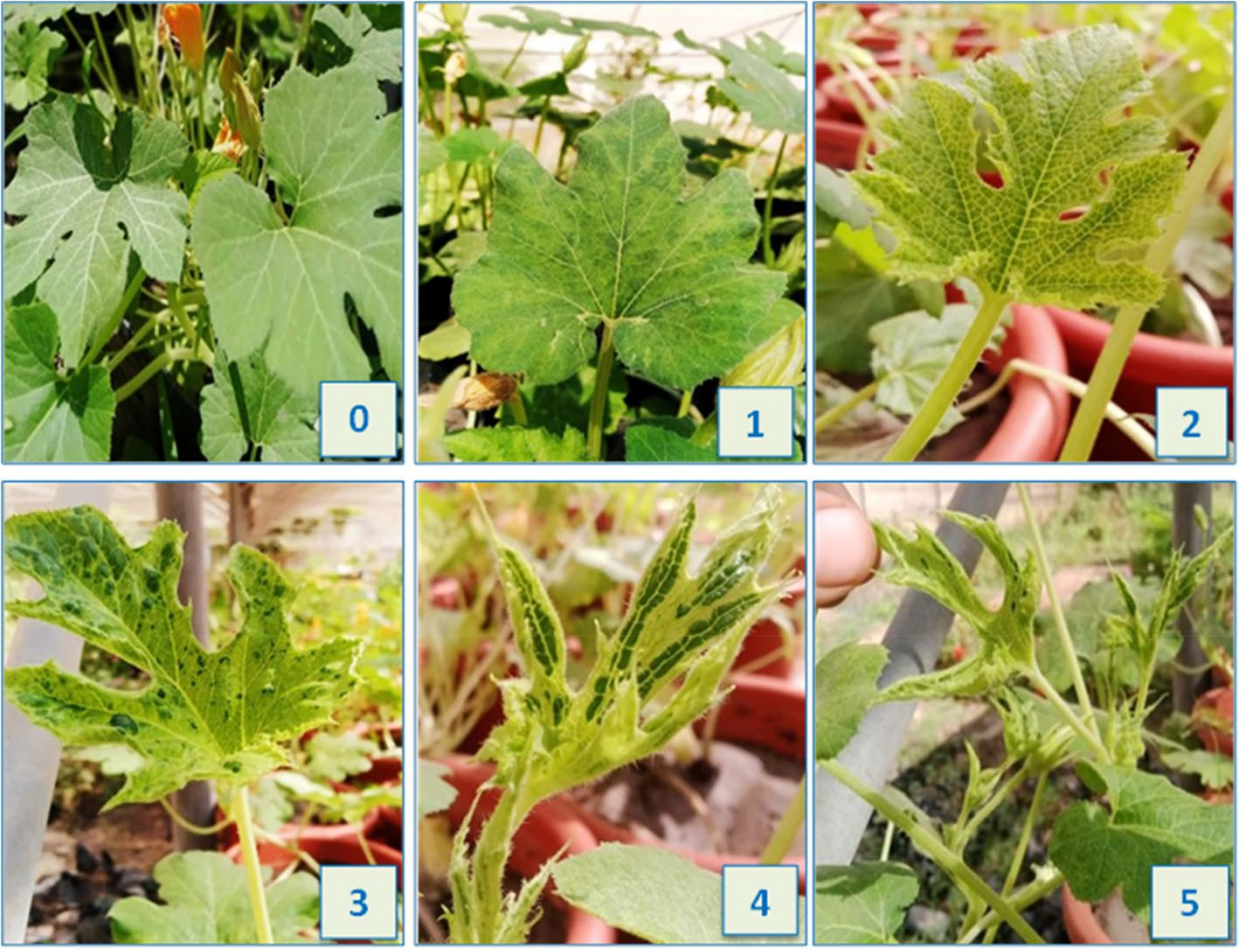
Disease severity index from 0 to 5, where 0 = no symptoms; 1 = very mild mosaic; 2 = mild yellow mosaic; 3 = yellowing and blistering; 4 = vein-banding, leaf deformation; 5 = severe mosaic, leaf deformation and stunting

The disease severity index (DSI) described the damage caused by ZYMV using a numerical scale of grades 0–5 (Fig. 2) and the disease severity percentage (DS%) was calculated using the following formula [29].

$$\mathrm{DS}\%=\frac{\sum\left(\mathrm{Disease}\;\mathrm{severity}\;\mathrm{grade}\;\times\;\mathrm{Number}\;\mathrm{of}\;\mathrm{plant}\;\mathrm{in}\;\mathrm{each}\;\mathrm{grade}\right)}{\mathrm{Total}\;\mathrm{number}\;\mathrm{of}\;\mathrm{tested}\;\mathrm{plants}\;\times\;\mathrm{highest}\;\mathrm{disease}\;\mathrm{severity}\;\mathrm{grade}}\times\;100$$

#### Extraction and determination of photosynthetic pigments

The contents of chlorophyll-*a* (chl *a*) and chlorophyll-*b* (chl *b*) and total carotenoids were extracted and assessed within 5 h of sample collection to prevent pigment degradation using an acetone-based spectrophotometric extraction method reported by Metzner et al. [50]. Five milliliters of 85% (*v/v*) acetone solution were utilized to homogenize about 100 mg of squash seedling leaf tissue. The extracted solution was centrifuged at 5000 × g for 5 min and the chl *a*, chl *b*, and total carotenoids concentrations in mg/ml were measured against an 85% acetone a blank by colorimetric reading the optical density (OD) at 663 (OD_663_), 644 (OD_644_), and 452 (OD_452_) nm, respectively, using a Shimadzu UV-160A spectrophotometer apparatus (Kyoto, Japan) applying the following formulas, and expressed as mg/g fresh weight (FW).

$$\mathrm{Chl}\;a=\;\left(10.3\;\times\;{\mathrm{OD}}_{663}\right)\;-\;\left(0.918\;\times\;{\mathrm{OD}}_{644}\right)$$

$$\mathrm{Chl}\;b=\;\left(19.7\;\times\;{\mathrm{OD}}_{644}\right)\;-\;\left(3.87\;\times\;{\mathrm{OD}}_{663}\right)$$

$$\mathrm{Total}\;\mathrm{carotenoids}\;=\;\left(4.2\;\times\;{\mathrm{OD}}_{452}\right)\;-\;\left(0.0264\;\mathrm{Chl}\;a\;+\;0.426\;\mathrm{Chl}\;b\right)$$

#### Malondialdehyde (MDA) quantification

Lipid peroxidation was assessed by quantifying the MDA level (μmol/g FW) as a biomarker of oxidative cell damage. MDA was assessed following the thiobarbituric acid (TBA) standard method as per Hodges et al. [51]. Fresh leaf samples (0.5 g) of squash were centrifuged at 12,000 × g at 4 °C for 15 min after being extracted with a 50 mM sodium phosphate buffer (pH 7.8). A 2 ml volume of the supernatant was gently vortexed with 2 ml of the 0.6% TBA containing 10% trichloroacetic acid (TCAA) before being heated in a water bath for 10 min. After cooling the mixed solution in an ice bath for 5 min, the mixture was centrifuged at 10,000 rpm for 5 min. MDA content was spectrophotometrically determined in μmol/g FW by measuring the OD at 532 (OD_532_), 600 (OD_600_), and 450 (OD_450_) nm using a spectrophotometer (Shimadzu UV-160A) and applying the following formula:

$$\mathrm{MDA}\;\left(\mu\mathrm{mol}/\mathrm g\;\mathrm{FW}\right)=\frac{\left[6.45\times\left({\mathrm{OD}}_{532}-{\mathrm{OD}}_{600}\right)-0.56\times{\mathrm{OD}}_{450}\right]\times\mathrm{Extract}\;\mathrm{volume}\;\left(\mathrm{ml}\right)}{\mathrm{Leaf}\;\mathrm{tissue}\;\mathrm{fresh}\;\mathrm{weight}\;\left(\mathrm g\right)}$$

#### Assay of enzymatic antioxidant activities

To prepare the enzyme extract, half a gram of fresh squash leaf tissue was homogenized in 10 mL of pre-chilled potassium phosphate extraction buffer (0.1 M, pH = 7.5, and containing 0.5 mM EDTA) using a cold mortar and pestle maintained strictly on ice to prevent enzyme degradation. The resulting homogenates were filtered and then centrifuged (15,000 × g for 15 min at 4 °C). The clear supernatant was collected for quantifying the ascorbate peroxidase (APX) [52], superoxide dismutase (SOD) [53], catalase (CAT) [54], glutathione reductase (GR) [55], and polyphenol oxidase (PPO) enzymes [56] using a UV–Vis spectrophotometer (Shimadzu Corp., UV-160, Kyoto, Japan). To calculate the specific enzyme activities, the total soluble protein concentration of each enzyme extract was first determined according to the Bradford method using bovine serum albumin as a standard [57]. The activities for all assessed enzymes were expressed as µmol/min/mg protein.

#### Assay of osmoregulators and non-enzymatic antioxidants

Using 0.1 g of dried and powdered plant material from the youngest and completely developed squash leaves, the total soluble sugars (mg/g dry weight; DW) were extracted with 5 mL of 80% (*v/v*) ethanol in a water bath at 80 °C for 30 min. Following centrifugation, the supernatant was collected, and the sugar content was spectrophotometrically determined in triplicate biological replicates following the ethanolic-anthrone procedure outlined in Irigoyen et al. [58]. The content of total soluble proteins (mg/g DW) in squash leaves was quantified following the Lowry-Folin standard method [59]. The total concentration of free proline (mg/g DW) was determined using the acidic ninhydrin spectrophotometry technique published by Bates et al. [60] utilizing L-proline to plot the standard curve and toluene as a blank reagent. The extraction and quantification protocols outlined in the Anderson [61] method were applied to assay the reduced glutathione (mg/g DW) content using 5, 5'-dithiobis (2-nitrobenzoic acid)-based GSH oxidation. As per Jagota and Dani [62], ascorbic acid (mg/g DW) content was extracted and spectrophotometrically quantified at 760 nm using a 5% TCAA solution (*v/v*) and a solution (1:10; *v/v*) of Folin-Ciocalteu reagent. The amounts of ascorbic acid in the samples were determined using a standard calibration curve with analytical grade ascorbic acid. The colorimetric approach detailed by Sauvesty et al. [63] was applied to extract and assess the total phenolics (mg/g DW) in dried squash leaves using a 70% ethanolic solution (*v/v*) at 40 °C overnight. The total phenolic concentrations in each sample were determined by deploying a standard curve for pyrogallol.

#### Squash growth characteristics

Five weeks after sowing, three randomly selected representative squash plants were collected from each of the five replicates (resulting in a total of 15 plants per treatment). The plants were carefully uprooted from the pots to avoid taproot damage and gently washed to remove adhering soil particles prior to growth measurements. Shoot length and root length were measured in cm using a meter scale and the leaf number/plant was counted. After registering the plant fresh weight (g), squash plants were oven-dried at 80 °C until they reached a constant weight, and their plant dry weights (g) were determined.

### Statistical analysis

The obtained data were further analyzed and plotted using Microsoft Excel® 2016 and the Genstat (11th edition, VSN International Ltd., Oxford, UK) software statistical package to evaluate the results of the present experiment. All the parameters were presented as means ± their standard errors (SEs). A two-way fixed-effects ANOVA (analysis of variance) was performed as a classical statistical option, followed by Duncan’s post-hoc test (*p* ≤ 0.05^*^ or *p* ≤ 0.01^**^; [64], to explore the statistical differences between the healthy or infected control squash plants and each of the other experimental groups treated with three different CS-CuO nanocomposites, each applied at three different concentrations. The experimental treatment factor was considered fixed, whereas the replication factor was considered random. The statistical R (version 4.0.2) software program was used for constructing the PCA biplot using the FactoMineR/factoextra packages, and Pearson’s correlation coefficient correlogram using the corrplot package among the variables selected.

## Results

### Virus isolation and identification

**Fig. 3:**
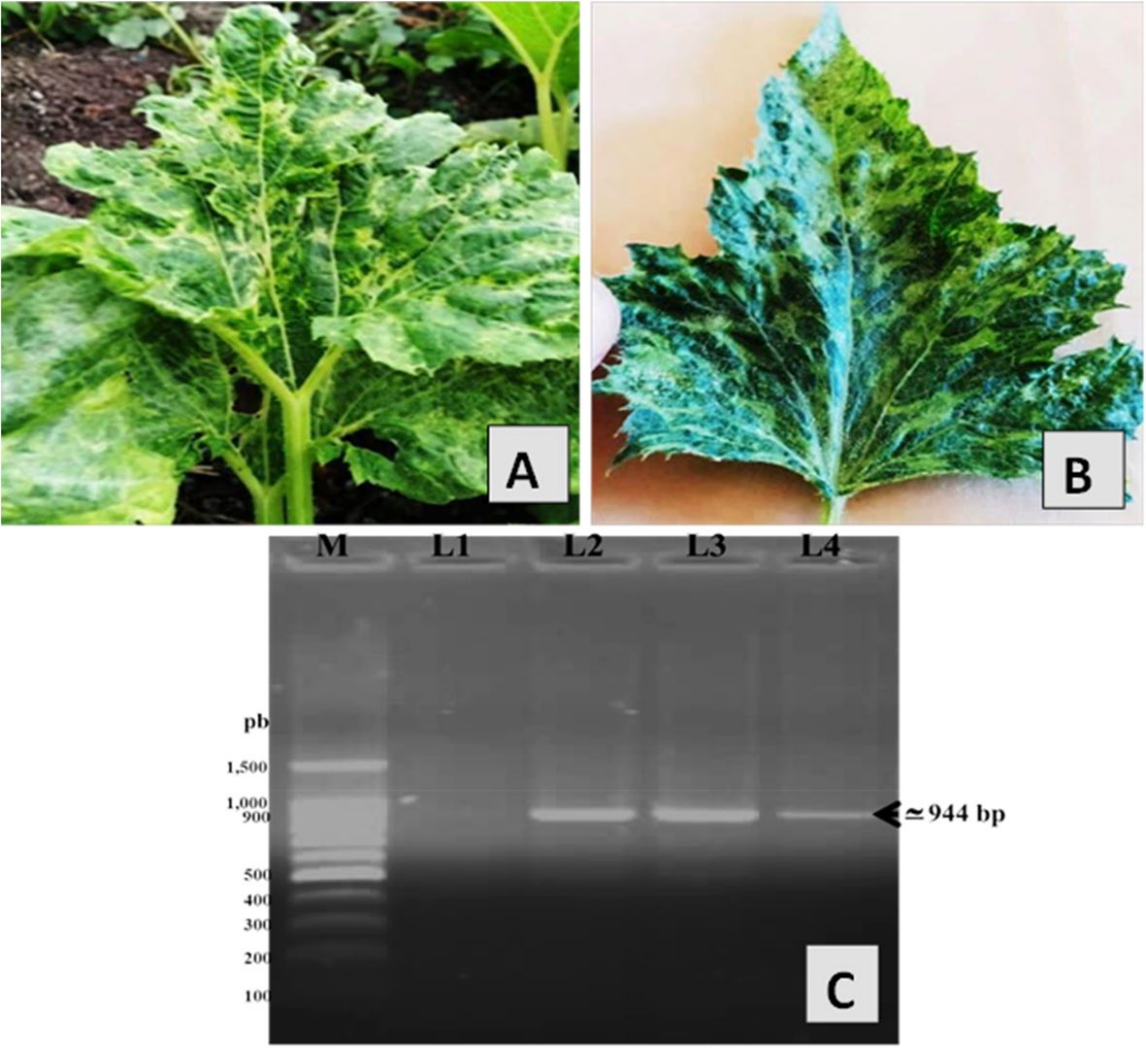
Symptoms of zucchini yellow mosaic virus (ZYMV) on naturally infected *C. pepo* include yellow mosaic green blisters, stunting, deformation, and reduction in leaf size (**A**), similar symptoms on ZYMV- mechanically inoculated squash plants (**B**), and electrophoretic gel (1% agarose) analysis of PCR products of ZYMV amplified with primers specifically for the P1 coding region. M: 100 bp DNA ladder. L1: negative control, L2: positive control, L3: Sample of the naturally infected squash leaves, L4: Samples of mechanically inoculated squash plants (**C**)

The naturally infected squash plants collected from open fields exhibited typical symptoms of ZYMV infection, including yellow mosaic, green blistering, leaf deformation, stunting, and reduced leaf size (Fig. 3A). The isolated pathogen induced identical visual symptoms upon mechanical inoculation into healthy squash seedlings (Fig. 3B). Because the P1 coding region represents one of the most highly variable genomic segments in potyviruses, it serves as an excellent molecular marker for strain differentiation, phylogenetic mapping, and precise taxonomic classification. Targeting this region facilitated the identification of genetic diversity and allowed us to accurately distinguish this local isolate from other global strains. The RT-PCR amplification using primers specific to the ZYMV P1-encoding region generated the expected 944 bp fragment in infected samples, whereas no amplification was detected in healthy control plants (Fig. 3C).

### Sequence analysis

**Fig. 4:**
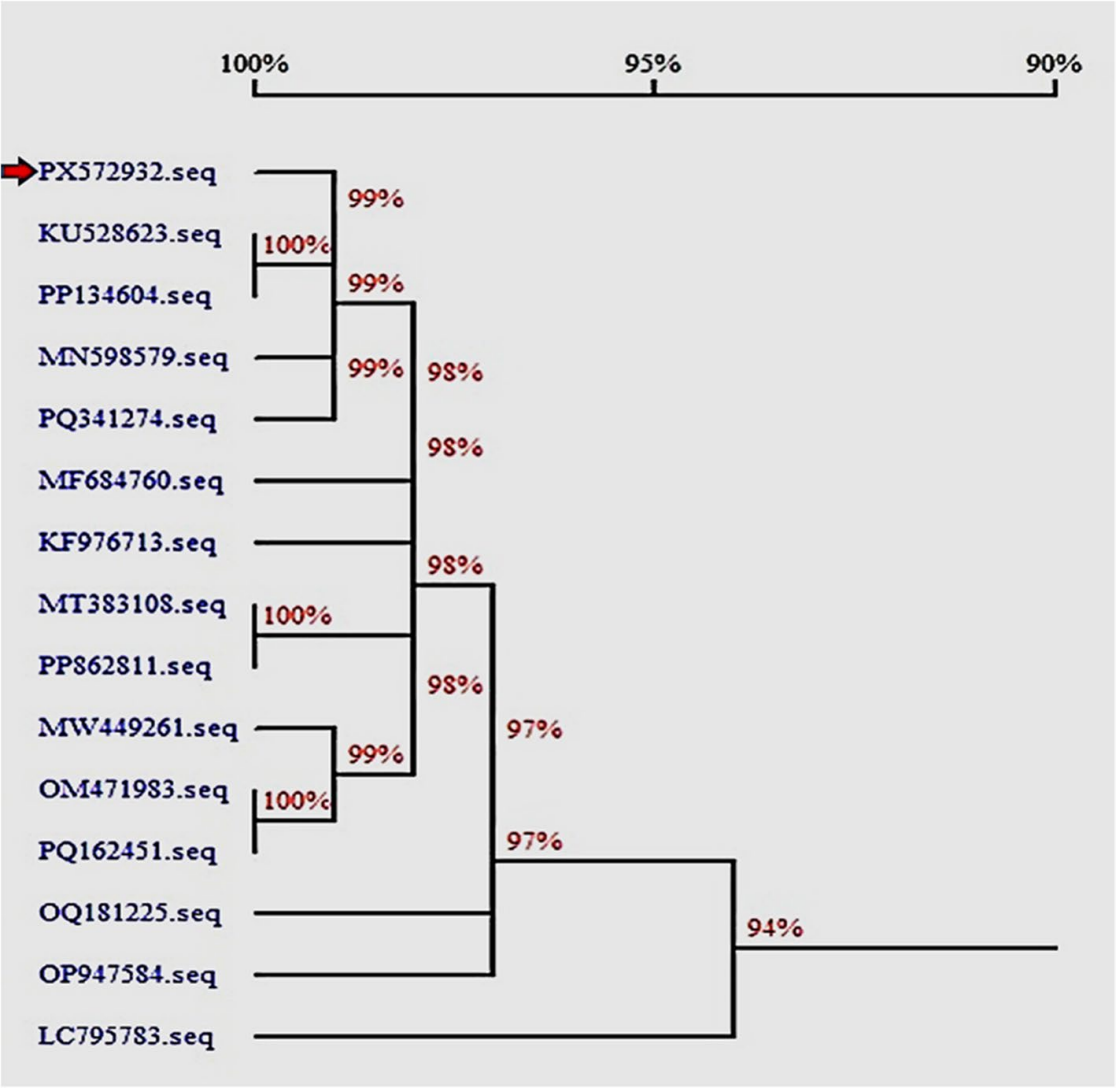
Phylogenetic tree of zucchini yellow mosaic virus showing the relationship between the Egyptian zucchini yellow mosaic virus (ZYMV) isolate (marked with red arrow) and 14 ZYMV sequences reported in GenBank using partial nucleic acid sequence of the P1 protein gene

The amplified P1 gene fragment of the Egyptian ZYMV isolate was sequenced and deposited in the NCBI GenBank as a partial sequence under accession number PX572932. Sequence analysis and homology comparisons were performed between the partial P1-encoding region of this local isolate and fifteen representative global ZYMV isolates registered in the database. A phylogenetic tree was subsequently constructed using homology-tree matrix analysis. Comparative sequence analysis with representative ZYMV isolates revealed high nucleotide identity ranging from 93.9% to 99.6% (Fig. 4; Table 2). The Egyptian isolate (PX572932) shared the greatest similarity with another Egyptian isolate (PP134604), whereas it exhibited the lowest sequence identity with isolate LC795783.

**Table 2:** Nucleotide identity of Egyptian zucchini yellow mosaic virus isolate with other isolates reported in GenBank to infect various hosts from different countries around the world

Accession number	Identity (%)	Host	Location
PP134604	99.6	Squash	Egypt
KU528623	99.4	Squash	Iran
PQ341274	98.5	Squash	Portugal
MN598579	98.4	melon	Australia
MF684760	98.2	Squash	Iran
OM471983	98.0	Squash	United Kingdom
MW449261	97.9	melon	France
PQ162451	97.6	cucumber	USA
KF976713	97.5	Squash	Slovakia
MT383108	96.7	Squash	Egypt
PP862811	96.7	Squash	Egypt
OP947584	96.5	cucumber	Cote d'Ivoire
OQ181225	96.4	Pumpkin	Peru
LC795783	93.9	Squash	Egypt

### Synthesis and characterization of chitosan-loaded copper oxide (CS-CuO) nanocomposite

The resulting TEM images provided quantitative morphological validation, revealing that the CSNPs displayed a thin, sheet-like structure with an average particle size of 10 ± 3.5 nm. Meanwhile, the uncoupled CuONPs exhibited a predominantly spherical morphology. The DLS and TEM dimensional analysis confirmed three distinct size categories for these synthesized CuONPs, yielding average diameters of 5 ± 1.8 nm, 20 ± 2.4 nm, and 50 ± 3.4 nm. Following the coupling reaction, these three size variants of CuONPs were individually immobilized on the CSNP surfaces in the presence of a stabilizing agent. The resulting nanocomposite containing the smallest CuONPs exhibited an irregular composite morphology with densely distributed dark spots (CuO) firmly anchored to the CSNP matrix, yielding a total average diameter of 17.18 ± 3.5 nm (Fig. 1A; designated hereafter as CS-CuO_17_). Similarly, nanocomposites synthesized with the medium and large CuONPs yielded correspondingly larger final structures, presenting quantitatively validated average sizes of 30.20 ± 2.5 nm (CS-CuO_30_) and 70.33 ± 1.2 nm (CS-CuO_70_), respectively.

### Effect of chitosan-loaded copper oxide (CS-CuO) nanocomposite on disease incidence and severity

**Fig. 5:**
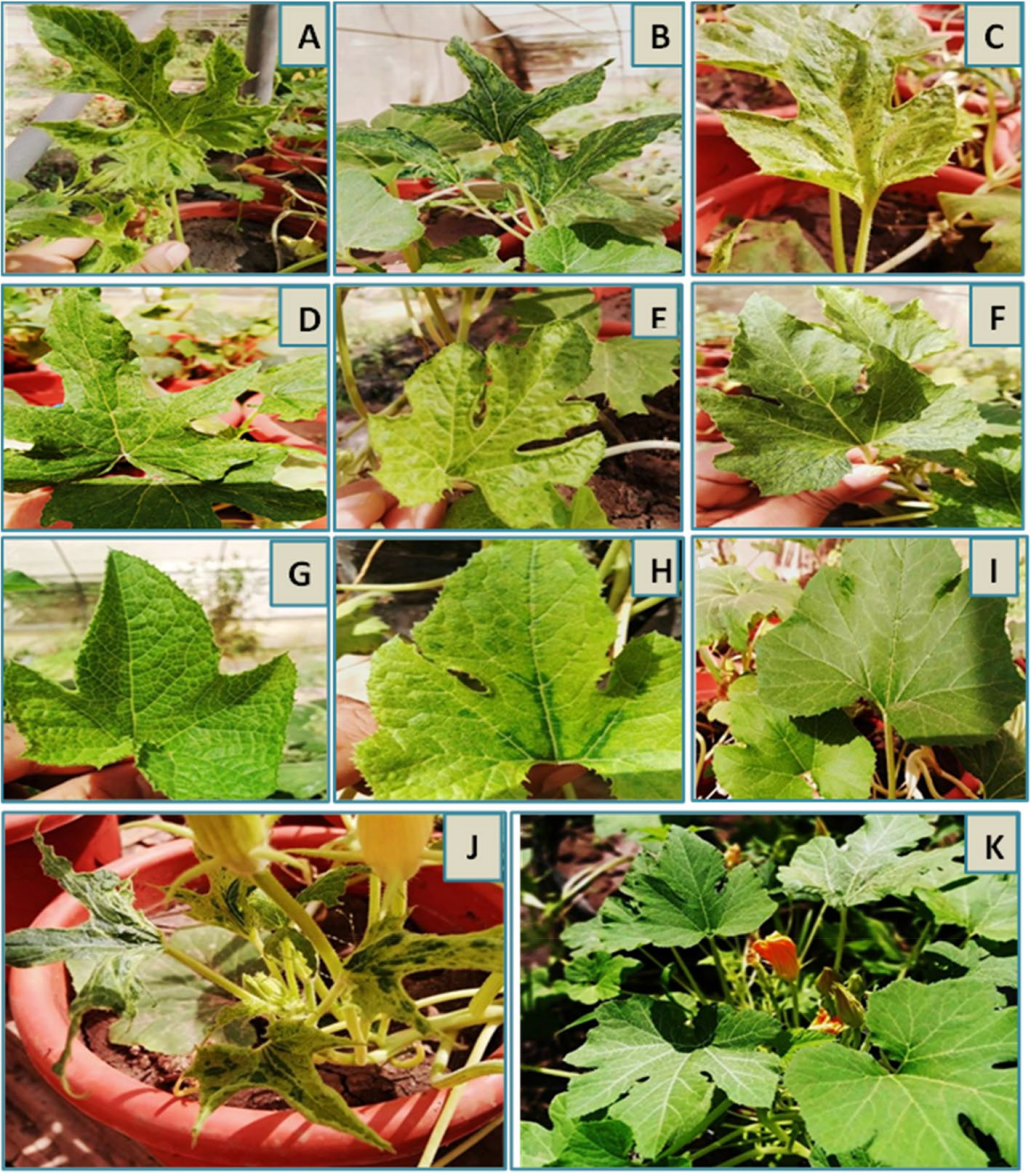
Effect of CS-CuO nanocomposites on the disease symptoms development on squash leaves infected with zucchini yellow mosaic virus (ZYMV) at 21 days post-inoculation. **A**, **B**, and **C** = plants sprayed with CS-CuO_70_ at 50,100 and 250 mg/L from left to right, respectively. **D**, **E**, and **F** = plants sprayed with CS-CuO_30_ at 50,100 and 250 mg/L from left to right, respectively. **G**, **H**, and **I** = plants sprayed with CS-CuO_17_ at 50,100 and 250 mg/L from left to right, respectively. **J** = ZYMV-inoculated plants (infected control). **K** = Mock-inoculated control plants (healthy control)

**Fig. 6:**
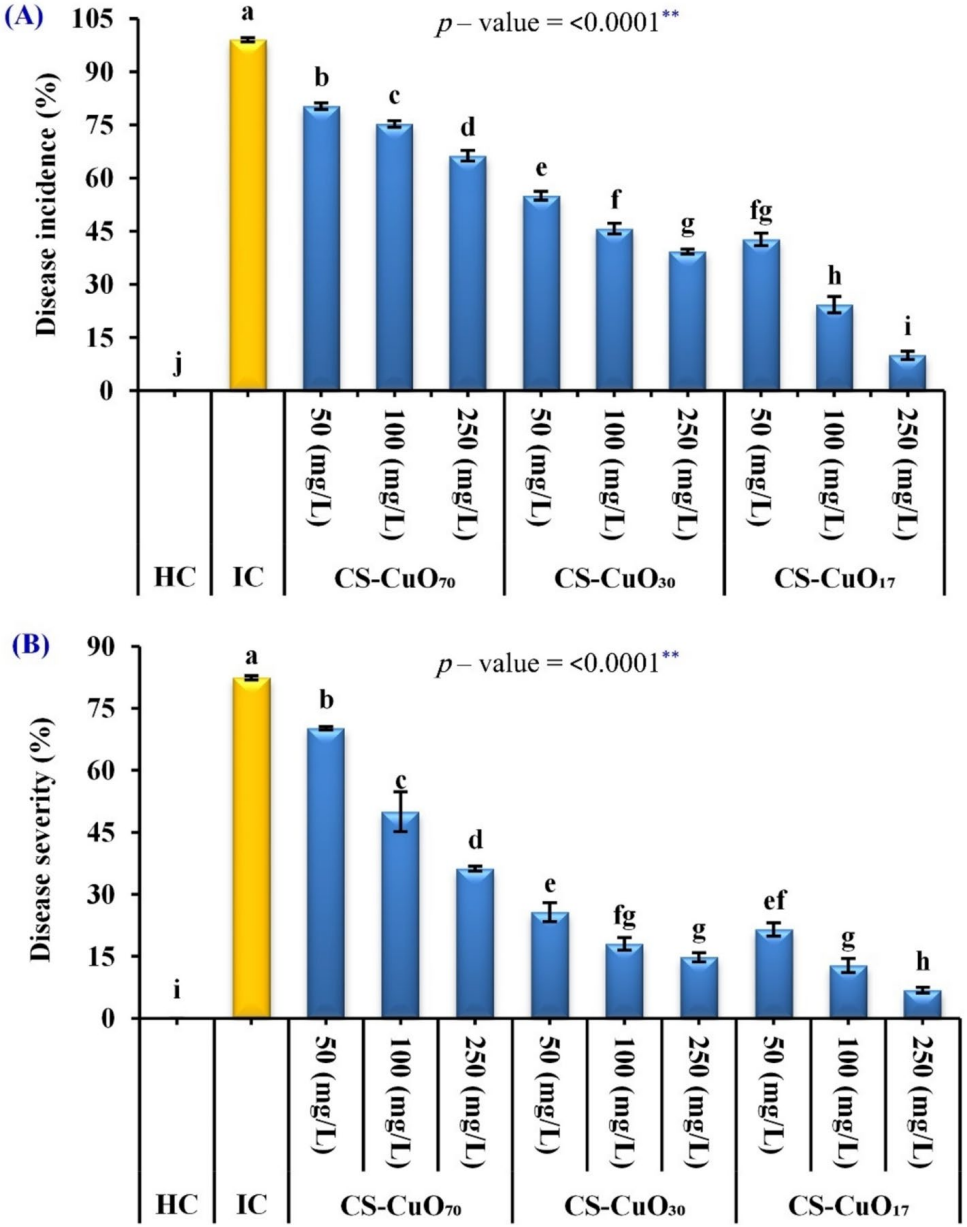
The impact of chitosan-loaded copper oxide (CS-CuO) nanocomposite foliar application on zucchini yellow mosaic virus (ZYMV) disease incidence (**A**) and disease severity (**B**). HC = healthy control (i.e., healthy plants sprayed with double-distilled water only), IC = infected control (i.e., untreated ZYMV-inoculated squash plants), and CS-CuO nanocomposite = nanocomposite of chitosan-loaded copper oxide (both in nano-form). Bars sharing the same letter do not differ significantly (*p* > 0.05) according to Duncan’s multiple range test. Asterisks indicate levels of significance (**p ≤ 0.01)

Severe mosaic, blistering, and stunting symptoms developed in untreated ZYMV-inoculated squash plants, whereas mock-inoculated plants remained symptomless (Fig. 5J, K) at 15 days post-inoculation under greenhouse conditions. Application of CS-CuO nanocomposites (48 h post-virus inoculation) as curative treatments reduced the percentage of ZYMV-infected plants. Foliar application of CS-CuO_17_ at 250 mg/L led to a substantial 89.9% reduction in ZYMV disease incidence, whereas CS-CuO_70_ at 50 mg/L achieved only an 18.9% reduction relative to untreated-infected squash control plants (Fig. 6A). Concerning disease severity, the results indicated that CS-CuO nanocomposites had varied protective impacts on symptom development, ranging from minor leaf distortions to completely asymptomatic phenotypes. Foliar application of CS-CuO_17_ at 250 mg/L led to the macroscopic remission of visible ZYMV symptoms (Fig. 5I) and significantly (*p* ≤ 0.05) reduced the calculated ZYMV disease severity index by 91.8% compared with the untreated-infected squash control (Fig. 6B). The ZYMV-infected squash plants treated with 250 mg/L of the CS-CuO₃₀ nanocomposite showed a positive response and exhibited mild symptoms (Fig. 5F) with a reduction in visual disease severity by 82.0%. However, squash plants sprayed with 50 mg/L of the CS-CuO70 nanocomposite showed the lowest reduction in disease severity (14.8%; Figs. 5A and 6B).

### Effect of chitosan-loaded copper oxide (CS-CuO) nanocomposite on photosynthetic pigments

**Fig. 7:**
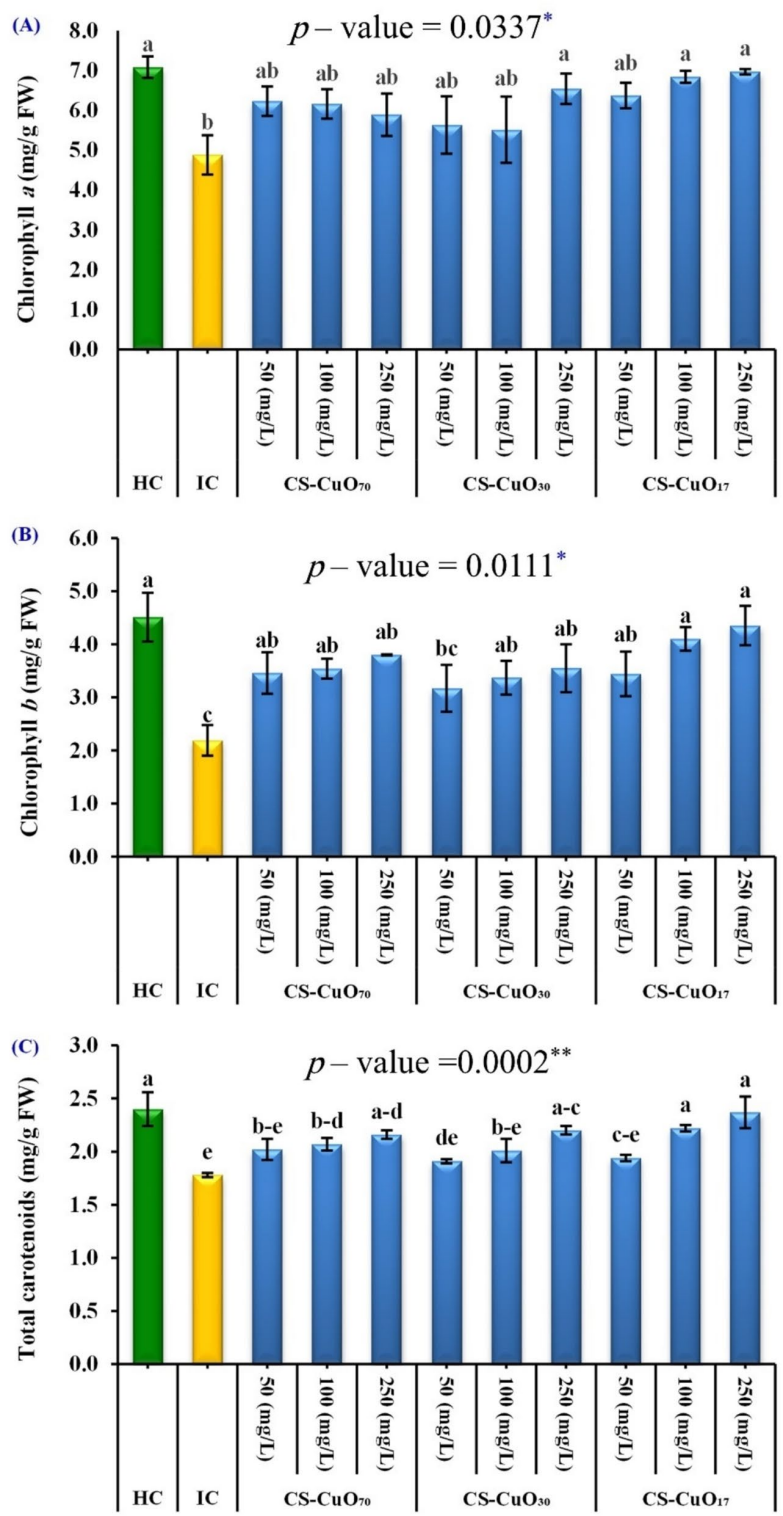
The impact of chitosan-loaded copper oxide (CS-CuO) nanocomposites foliar application on photosynthetic pigments (i.e., **A** Chlorophyll *a*, **B** Chlorophyll *b*, and **C** Total carotenoids) under zucchini yellow mosaic virus (ZYMV) infection. HC = healthy control (i.e., healthy plants sprayed with double-distilled water only), IC = infected control (i.e., untreated ZYMV-inoculated squash plants), and CS-CuO nanocomposite = nanocomposite of chitosan-loaded copper oxide (both in nano-form). FW = Fresh weight. Bars sharing the same letter do not differ significantly (*p* > 0.05) according to Duncan’s multiple range test. Asterisks indicate levels of significance (*p ≤ 0.05 and **p ≤ 0.01)

A substantial reduction in the photosynthetic pigments of squash leaves was noted in untreated ZYMV-infected control. Chlorophyll *a*, chlorophyll *b*, and total carotenoids decreased by 45.1%, 105.9%, and 34.8%, respectively, in untreated infected plants displaying severe mosaicism and viral symptoms compared to healthy controls (Fig. 7A–C). However, foliar application of CS-CuO nanocomposites significantly (*p* ≤ 0.05) alleviated this virus-induced decline, prompting substantial increases in all pigment fractions. Across all CS-CuO formulations and concentrations, chlorophyll *a* content increased by 12.9% to 42.8%, chlorophyll *b* by about 44.7% to 98.6%, and total carotenoids by 7.3% to 33.1% relative to untreated ZYMV-inoculated plants. Notably, the CS-CuO_17_ formulation exhibited the most consistent and pronounced improvements, particularly at 100 and 250 mg L^−1^, where chlorophyll *a* increased by around 40.2% to 42.8%, chlorophyll *b* by 87.2% to 98.6%, and total carotenoids by 24.7% to 33.1% compared with the untreated-infected control plants (Fig. 7A-C).

### Effect of chitosan-loaded copper oxide (CS-CuO) nanocomposite on membrane lipid peroxidation and enzymatic antioxidant activities

**Fig. 8:**
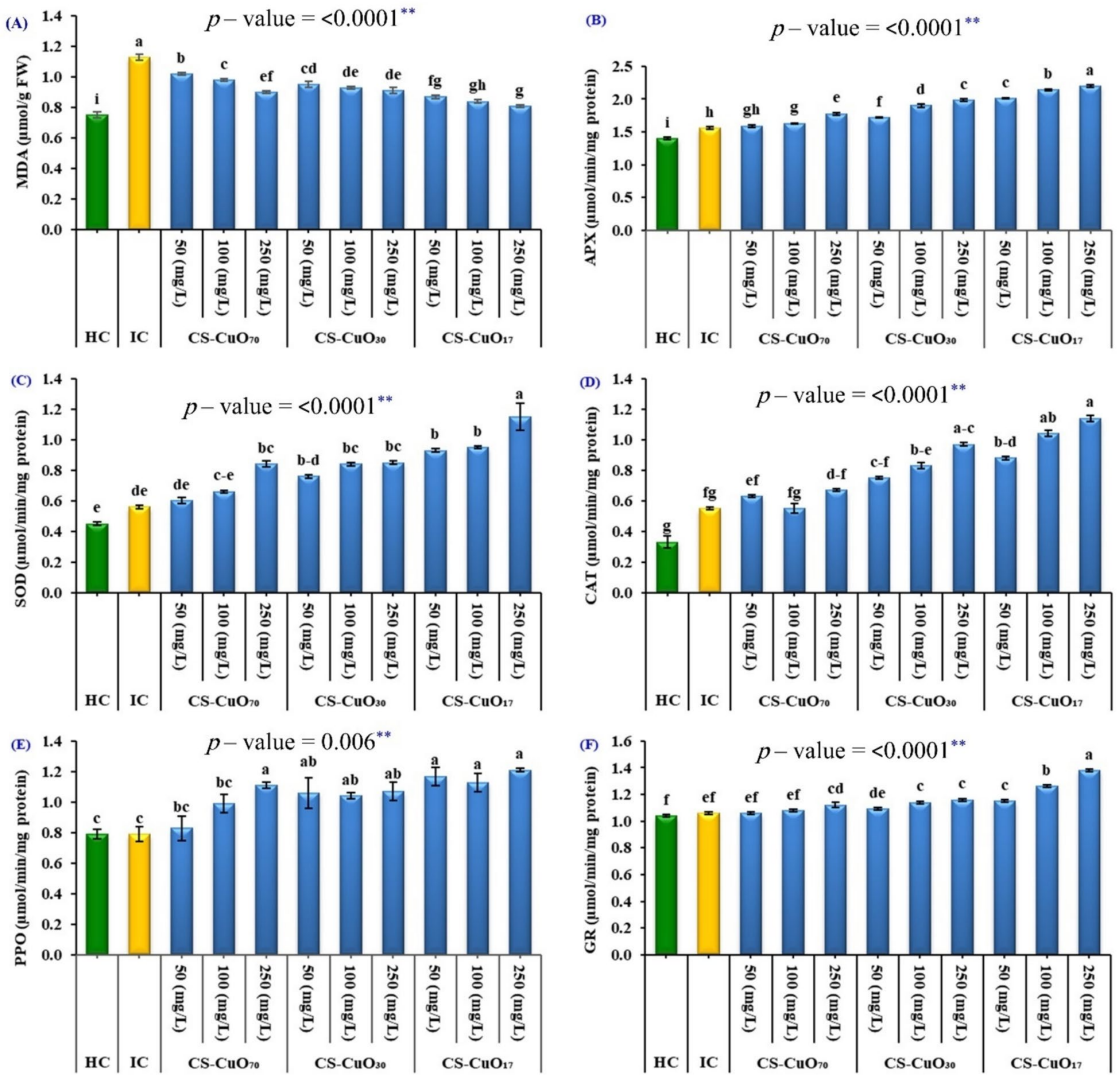
The impact of chitosan-loaded copper oxide (CS-CuO) nanocomposite foliar application on (**A**) malondialdehyde (MDA) content and activity of enzymatic antioxidants, e.g., **B** Ascorbate peroxidase (APX), **C** Superoxide dismutase (SOD), **D** Catalase (CAT), **E** Polyphenol oxidase (PPO), and **F** Glutathione reductase (GR) of *Cucurbita pepo* L. cv. Eskandarani plants inoculated with zucchini yellow mosaic virus (ZYMV) at 21 days post-inoculation. HC = healthy control (i.e., healthy plants sprayed with double-distilled water only), IC = infected control (i.e., untreated ZYMV-inoculated squash plants), and CS-CuO nanocomposite = nanocomposite of chitosan-loaded copper oxide (both in nano-form). FW = Fresh weight. Bars sharing the same letter do not differ significantly (*p* > 0.05) according to Duncan’s multiple range test. Asterisks indicate levels of significance (**p ≤ 0.01)

The malondialdehyde (MDA) level serves as a well-established biomarker for membrane lipid peroxidation in plant cells. The data presented in Fig. 8A demonstrate that foliar application of CS-CuO nanocomposites markedly lowered MDA accumulation in ZYMV-stressed leaves. A considerable decrease in MDA content by 20.4%, 19.5%, and 28.3% was observed when squash leaves were sprayed with the CS-CuO_70_, CS-CuO_30_, or CS-CuO_17_ nanocomposite at 250 mg/L compared to the ZYMV-infected control plants, respectively. The activities of antioxidant enzymes were significantly (*p* ≤ 0.05) upregulated in all the squash plant sets subjected to foliar sprays of CS-CuO nanocomposites at different applied concentrations. The obtained results showed that antioxidant enzymes, i.e., APX, SOD, CAT, PPO, and GR were boosted, and the highest activities were observed in squash plants treated with the CS-CuO_17_ nanocomposite at 250 mg/L, where their levels were 41.0%, 105.4%, 107.3%, 53.2%, and 30.2% higher than the untreated infected control plants, respectively (Fig. 8B-F). Interestingly, PPO activity under most nanocomposite treatments was statistically comparable to that of the 250 mg/L CS-CuO_17_ group, with minor exceptions observed only at the lowest tested concentrations (50 and 100 mg/L of CS-CuO_30_ and CS-CuO_70_). Furthermore, no significant (*p* > 0.05) differences in leaf CAT activity were detected between the 250 mg/L CS-CuO_17_, 250 mg/L CS-CuO_30_, and 100 mg/L CS-CuO_17_ treatment groups.

### Effect of chitosan loaded copper oxide (CS-CuO) nanocomposite on the accumulation of osmoregulators and non-enzymatic antioxidants

**Fig. 9:**
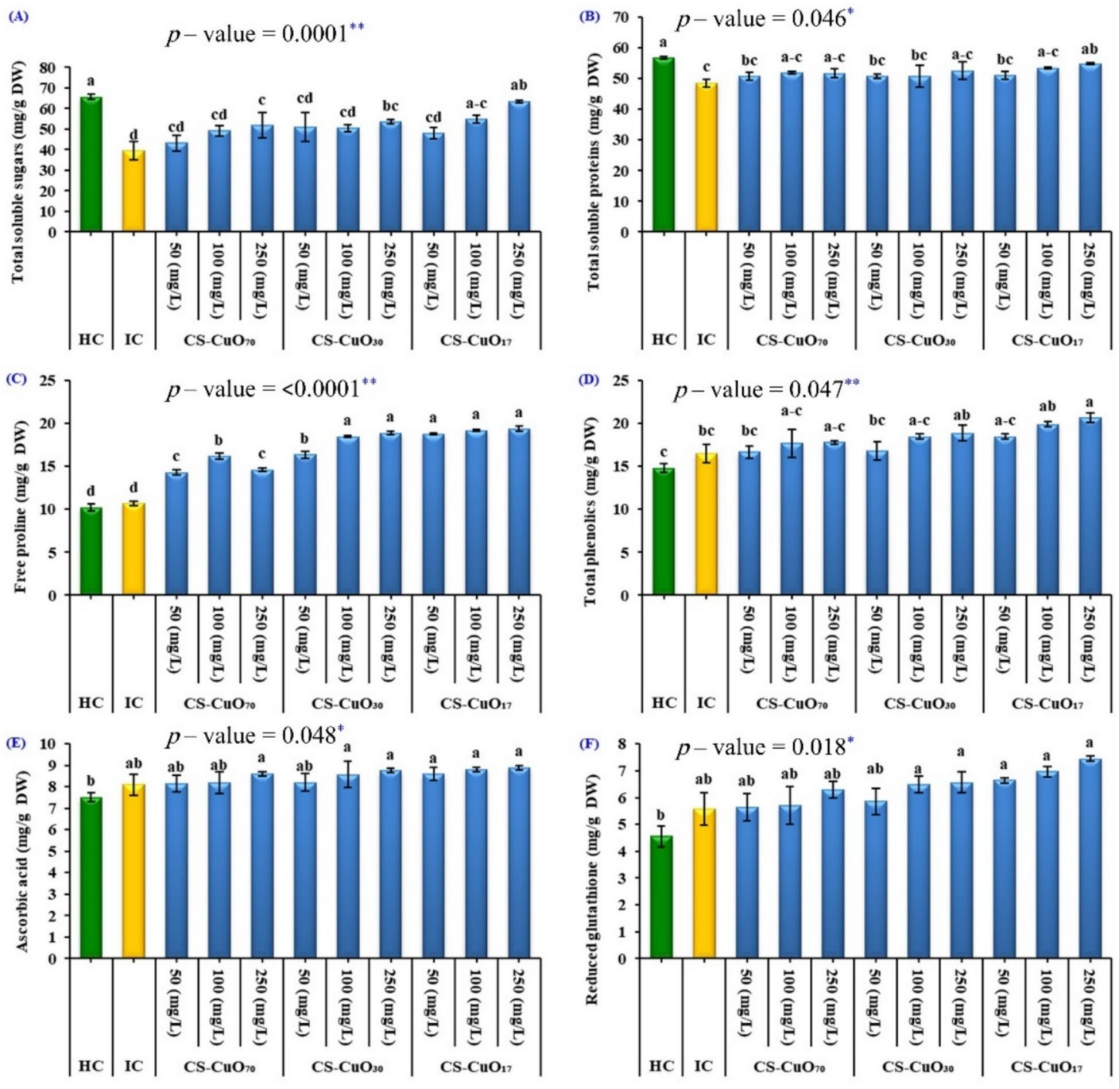
The impact of chitosan-loaded copper oxide (CS-CuO) nanocomposite foliar application on osmoregulatory compounds (i.e., **A** Total soluble sugars, **B** Total soluble proteins, and **C** Free proline) and non-enzymatic antioxidants (i.e., **D** Total phenolics, **E** Ascorbic acid, and **F** Reduced glutathione) of *Cucurbita pepo* L. cv. Eskandarani plants inoculated with zucchini yellow mosaic virus (ZYMV) at 21 days post-inoculation. HC = healthy control (i.e., healthy plants sprayed with double-distilled water only), IC = infected control (i.e., untreated ZYMV-inoculated squash plants), and CS-CuO nanocomposite = nanocomposite of chitosan-loaded copper oxide (both in nano-form). DW = Dry weight. Bars sharing the same letter do not differ significantly (*p* > 0.05) according to Duncan’s multiple range test. Asterisks indicate levels of significance (*p ≤ 0.05 and **p ≤ 0.01)

The impacts of ZYMV infection and subsequent CS-CuO nanocomposite treatments on leaf osmoregulators and non-enzymatic antioxidants at 21 dpi are presented in Fig. 9. In response to the ZYMV inoculation, the untreated ZYMV-infected squash plant leaves showed a significant (*p* ≤ 0.05) decline in total soluble sugars and total soluble proteins by 39.8% and 14.4%, respectively, compared to healthy controls. However, foliar application of CS-CuO nanocomposites, particularly CS-CuO_17_, at the different tested concentrations raised the total soluble sugars and total soluble proteins compared to the infected control plants. Exogenous application of CS-CuO_17_ nanocomposite at 250 mg/L, followed by CS-CuO_17_ nanocomposite at 100 mg/L and CS-CuO_30_ nanocomposite at 250 mg/L significantly (*p* ≤ 0.05) increased total soluble sugars by 60.5%, 38.7%, and 35.7% (Fig. 9A) and total soluble proteins by 13.0%, 10.1%, and 8.2%, respectively, compared to the untreated ZYMV-infected control plants (Fig. 9B).

In untreated infected leaf tissues, free proline, total phenolics, ascorbic acid, and reduced glutathione did not increase significantly (*p* > 0.05) compared to healthy controls, though minor upward stress-response trends were observed (Fig. 9C–F). Foliar application of the novel CS-CuO nanocomposites enabled the plants to effectively rebound from these pathological impacts. Following treatment, these cellular physio-biochemical traits gradually rose across all applied concentrations. The most prominent increases, reaching up to 76.6%–81.3% for free proline (Fig. 9C), 14.5%–25.5% for total phenolics (Fig. 9D), 6.2%–9.9% for ascorbic acid (Fig. 9E), and 17.5%–33.3% for reduced glutathione (Fig. 9F), were recorded in the groups treated with the 100 or 250 mg/L concentrations of CS-CuO_17_ and CS-CuO_30_, respectively, compared to the untreated ZYMV-infected control plants.

### Effect of chitosan-loaded copper oxide (CS-CuO) nanocomposite on squash growth characteristics

**Fig. 10:**
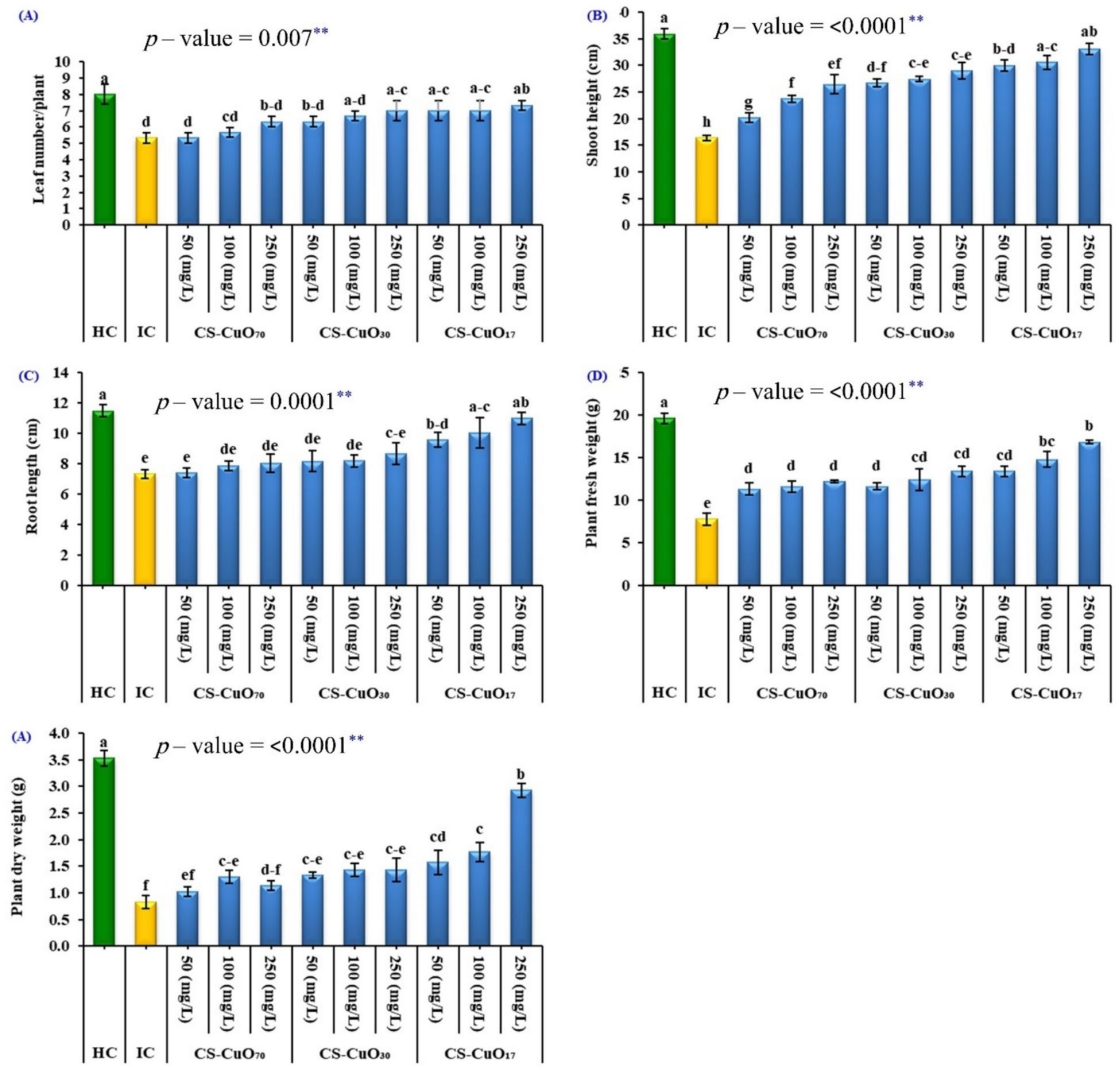
The impact of chitosan-loaded copper oxide (CS-CuO) nanocomposite foliar application on growth characteristics (i.e., **A** Leaf area/plant, **B** Shoot height, **C** Root length, **D** Plant fresh weight, and **E** Plant dry weight) in squash plants inoculated with zucchini yellow mosaic virus (ZYMV) at 21 days post-inoculation. HC = healthy control (i.e., healthy plants sprayed with double-distilled water only), IC = infected control (i.e., untreated ZYMV-inoculated squash plants), and CS-CuO nanocomposite = nanocomposite of chitosan-loaded copper oxide (both in nano-form). Bars sharing the same letter do not differ significantly (*p* > 0.05) according to Duncan’s multiple range test. Asterisks indicate levels of significance (**p ≤ 0.01)

In response to ZYMV inoculation, the leaf number/plant, shoot height, root length, plant fresh weight, and plant dry weight were severely suppressed by 33.4%, 54.3%, 36.1%, 60.2%, and 76.5%, respectively, compared to healthy controls (Fig. 10A–E). Conversely, foliar application of the CS-CuO_17_ nanocomposite at 100 or 250 mg/L proved to be the most effective treatment, stimulating these respective growth characteristics by 31.3%–37.5%, 86.6%–101.8%, 36.8%–50.1%, 89.7%–115.4%, and 113.3%–253.0% compared to the untreated infected plants.

### Relationship among implemented treatments and investigated traits

**Fig. 11:**
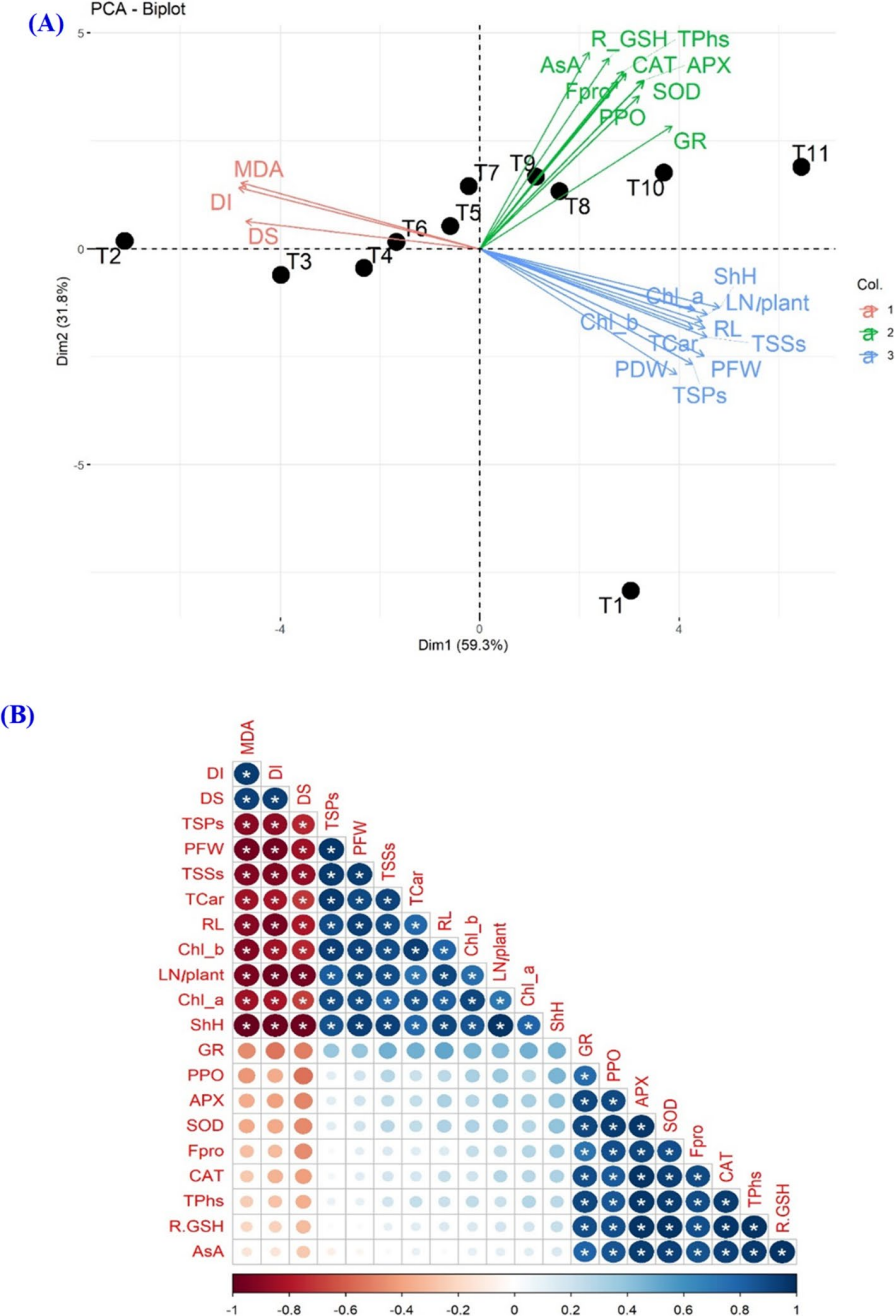
Principal component (PCA)-biplot (**A**) for applied treatments and studied traits. Pearson’s correlation coefficients, among the squash attributes studied, are shown in the correlogram (**B**). The circle color in the correlogram corresponds to the correlation coefficient, wherein a positive correlation coefficient is closer to 1 (purple end of the scale) and a negative correlation coefficient is closer to −1 (red end of the scale). The circle size matches the significance level. (*) indicates a significant (*p* ≤ 0.05) correlation. T1: HC = healthy control; T2: IC = infected control; T3: zucchini yellow mosaic virus (ZYMV)-infected squash plants sprayed with CS-CuO_70_ at 50 mg/L; T4: ZYMV-infected squash plants sprayed with CS-CuO_70_ at 100 mg/L; T5: ZYMV-infected squash plants sprayed with CS-CuO_70_ at 250 mg/L; T6: ZYMV-infected squash plants sprayed with CS-CuO_30_ at 50 mg/L; T7: ZYMV-infected squash plants sprayed with CS-CuO_30_ at 100 mg/L; T8: ZYMV-infected squash plants sprayed with CS-CuO_30_ at 250 mg/L; T9: ZYMV-infected squash plants sprayed with CS-CuO_17_ at 50 mg/L; T10: ZYMV-infected squash plants sprayed with CS-CuO_17_ at 100 mg/L; T11: ZYMV-infected squash plants sprayed with CS-CuO_17_ at 250 mg/L. DS: disease severity; DI: disease incidence; Chl *a*: chlorophyll *a*; Chl *b*: chlorophyll *b*; TCar: total carotenoids; MDA: malondialdehyde; APX: ascorbate peroxidase; SOD: superoxide dismutase, CAT: catalase, PPO: polyphenol oxidase, GR: glutathione reductase; TSSs: total soluble sugars; TSPs: total soluble proteins; Fpro: free proline; TPhs: total phenolics; AsA: ascorbic acid; R-GSH: reduced glutathione; LN/plant: leaf number/plant; ShH: shoot height; RL: root length; PFW: plant fresh weight; PDW: plant dry weight. Col.: color. Each black bubble (•) refers a treatment number

A principal component analysis (PCA) biplot accounted for 91.1% of the total variance across the first two principal components (Fig. 11A), clearly segregating the experimental groups based on nanocomposite size and application concentration. Treatments involving smaller-sized nanocomposites, particularly CS-CuO_17_ at 100 and 250 mg/L, clustered closely with growth traits, photosynthetic pigments, and osmoprotective compounds, indicating superior protective efficacy against ZYMV stress. In contrast, oxidative stress-related attributes showed negative associations with plant growth and productivity parameters. Overall, the PCA confirmed the strong integrative role of CS-CuO_17_ in enhancing physiological performance and stress tolerance in ZYMV-infected squash plants. Plant fresh weight displayed strong, significant positive correlations (*r* = 0.881 to 0.968, *p* ≤ 0.01; Fig. 11A, B) with vegetative vegetative shoot–root attributes, photosynthetic pigments, and osmoregulatory compounds (total soluble sugars and proteins). Conversely, fresh weight exhibited a strong, negative correlation (*r* = −0.840 to −0.952, *p* ≤ 0.01) with disease parameters (incidence and severity) and the oxidative stress marker MDA. Meanwhile, correlations between plant fresh weight and both enzymatic and non-enzymatic antioxidant pools remained weak and statistically non-significant (*p* > 0.05).

## Discussion

ZYMV is among the most serious viral diseases of economically important cucurbit crops, causing significant fruit yield losses in infected plants, especially in semi-arid regions worldwide [65]. Although higher plants lack an adaptive immune response against both abiotic and biotic stress, they can nonetheless develop an adaptive or tolerance defense mechanism [66]. The successful amplification of the ~ 944bp P1 gene confirms the presence of ZYMV, aligning with previous molecular detections [28]. Furthermore, phylogenetic comparisons of the P1 gene validate the isolate’s identity, showing a high evolutionary conservation (93.9–99.6% identity) with local Egyptian strains, likely driven by regional vector dynamics, shared environmental conditions, and the localized exchange of infected seeds [23]. The robust curative potential demonstrated by the CS-CuO_17_ formulation is mechanistically driven by the dual capability of CSNPs to inhibit plant viruses via direct antiviral action and by triggering endogenous plant defense mechanisms [29, 32]. The positively charged amino groups of CSNPs likely facilitate direct attachment to the negatively charged phosphate groups of viral RNA or the glycol proteins of the viral coat, thereby inhibiting viral replication and suppressing symptom development [29, 67, 68].

The manifestation of virus-induced chlorotic and pathological symptoms inherently reflects disturbances in photosynthetic pigment formation [67, 69], alterations in enzymatic antioxidant activities, and fluctuations in endogenous non-enzymatic metabolites [70]. Because ZYMV multiplication and mobility exploit chloroplast factors, the virus adversely affects chloroplast stability, disrupting photosystem II complexes and causing severe chlorosis [71]. The observed reduction in chlorophylls and carotenoids during infection is likely driven by the overproduction of reactive oxygen species (ROS) at the chloroplast, leading to the destruction of photosynthetic pigments and a subsequent collapse in carbon dioxide fixation [68, 72, 73]. Nanocomposites, particularly those under 100 nm, exhibit enhanced reactivity due to their immense surface area-to-volume ratios [29]. Because plant viruses are nanoscale biological entities, the CS-CuO nanocomposites likely facilitate direct antiviral action by intercepting viral particles and disrupting their replication within host tissues [25]. The superior performance of the CS-CuO_17_ formulation may stem from its capacity to alter the structural integrity of the virus, promoting the formation of faulty viral particles. This interaction is mediated by the high surface area and the positively charged amino (− NH2) functional groups of the glucosamine polymer chains [74, 75], which electrostatically bind viral mRNA and carboxyl (− COO-) groups of viral amino acids [76], thereby arresting DNA replication. Beyond the physical disruption of the virus, the elemental copper supplied by the nanocomposites plays a crucial physiological role. Copper is fundamental to electron transport during the light reactions of photosynthesis as a core component of plastocyanin, the most abundant Cu-containing protein in chloroplasts [77]. Furthermore, it uniquely functions within the plant cellular system as a vital cofactor for several antioxidant enzymes via redox reactions and photophosphorylation [78]. In conjunction with these Cu-driven processes, the restoration of photosynthetic pigments is fundamentally linked to the nanocomposite's ability to enhance nutrient assimilation, specifically nitrogen and Mg [79], which are fundamental building blocks for the chlorophyll molecule and vital photosynthetic enzymes [80, 81]. Furthermore, the released CuO can restrict infectivity by physically blocking viral contact with live host cells [82], while the CSNP matrix prevents cell-to-cell systemic spread by promoting callose precipitation at plasmodesmata and inhibiting callose-degrading enzymes like β−1,3-glucanase [83, 84].

Viral stress triggers the overproduction of ROS (e.g., superoxide anion; O_2_^•−^, hydrogen peroxide; H_2_O_2_, and hydroxyl radical; OH^−^), culminating in severe cellular dysfunction [32, 85]. The nanocomposite’s capacity to halt membrane lipid peroxidation, evidenced by the stabilization of MDA levels (Fig. 8), further underscores its role in averting the bleaching of photosynthetic pigments via co-oxidation by lipid peroxy-radicals and H_2_O_2_, physically manifesting as mosaic and yellowing symptoms [86, 87]. This rapid alleviation of oxidative stress indicates that although bare metal oxide nanoparticles can induce free radical formation and subsequent oxidative cellular damage [32, 84], the applied CS-CuO formulations did not exhibit such phytotoxicity [88]. This protective advantage is likely due to the chitosan matrix, which acts as a stabilizing capping agent to buffer direct CuO toxicity while synergistically enhancing the plant's endogenous ROS-scavenging capacity [29, 67, 88]. Consequently, the CS-CuO nanocomposite structure indirectly regulates physio-biochemical attributes by altering water uptake [89], upregulating pathogen-responsive genes [67], and triggering defense mechanisms to fend off the oxidative burst [90].

The observed upregulation of antioxidant enzymes (APX, SOD, CAT, PPO, and GR) during viral stress reflects the host plant’s attempt to restore redox homeostasis, aligning with established defense responses in cucurbits [18, 68]. SOD provides the initial shield by converting highly toxic O_2_^•−^ into H_2_O_2_ [91], which is subsequently scavenged by APX and CAT [92]. GR regulates the glutathione pool via the Halliwell-Asada cycle [93]. Beyond acting as an oxygen reservoir and facilitating the water-to-water cycle during photosynthesis [94], PPO scavenges cellular ROS and promotes the synthesis of lignin and suberin to physically obstruct pathogen advancement [95, 96]. The specific amplification of these enzymatic cascades following CS-CuO_17_ nanocomposite application shifts the paradigm from mere antiviral action to active immunological modulation [90]. The released CSNPs act as elicitors, stimulating nitric oxide (NO) accumulation to modulate gene regulation and heavily upregulate detoxification enzymes [90, 97, 98], while the CuONPs likely upregulate the transcription of pathogenesis-related genes to obstruct plasmodesmatal diffusion [25].

The buildup of osmoregulatory compounds and non-enzymatic antioxidants plays a vital role in the plant’s defense capacity and ROS detoxification during viral infection [68, 99]. In untreated ZYMV-infected plants, however, the observed depletion of total soluble sugars and proteins positively correlates with the reduction in photosynthetic pigments and overall growth suppression [18]. Because cellular soluble sugars act as critical signaling pathways for nutrient elements and biometabolites that trigger hormonal crosstalk [100], their depletion severely disrupts defense-related gene expression [90]. This metabolic decline is likely exacerbated by restricted orthophosphate availability, which hinders the transport of photoassimilates from the chloroplast to the cytoplasm [101]. The CS-CuO-mediated induction of these osmoregulatory networks directly counteracts this metabolic decline by reprogramming protein biosynthesis and assembly [102]. Furthermore, the liberated CSNPs can act as metabolic intermediates, entering the tricarboxylic acid cycle to directly drive the biosynthesis of critical amino acids [103]. This includes the induction of proline biosynthesis and the restriction of its oxidation to L-glutamate [104]. Consequently, the accumulation of free proline preserves cellular turgidity, stabilizes membranes, and detoxifies ROS to mitigate virus-induced oxidative injury [70, 91]. While untreated infected plants exhibited a slight, passive increase in non-enzymatic antioxidants (phenolics, ascorbic acid, and reduced glutathione) in response to oxidative damage [18, 25, 105], the CS-CuO treatments actively and robustly amplified these pools. This enhancement points to the upregulation of secondary metabolic pathways, notably through the activation of phenylalanine ammonia-lyase [106]. The resulting surge in phenolic content decreases membrane fluidity to restrict free radical penetration [107], while the elevated ascorbic acid and glutathione actively feed the Halliwell-Asada cycle to reinforce overall cellular immunity [108].

Ultimately, the macroscopic rescue of vegetative growth is the direct phenotypic manifestation of these orchestrated biochemical and structural defenses [87, 108]. These results demonstrate that the unique physicochemical traits of CS-CuO nanocomposites (e.g., high reactivity, surface charge, and optimized size; Fig. 4) are highly effective at restoring both the biochemical and physical vitality of the host. These restorative mechanisms cumulatively reversed the virus-induced stunting, leading to significant improvements in shoot height, leaf surface area, root length, and total plant biomass (Fig. 10). While the current study comprehensively demonstrates the significant phenotypic, physiological, and morphological recovery of ZYMV-infected plants treated with CS-CuO nanocomposites. Future studies incorporating absolute viral load quantification via qRT-PCR will be highly informative to further elucidate the precise molecular dynamics of this viral replication inhibition.

## Conclusion

Following the molecular identification of the local ZYMV isolate, this study successfully synthesized size-variant chitosan-loaded copper oxide nanocomposites to evaluate their antiviral and protective potential. The findings reveal that these nanocomposites do not merely suppress viral symptoms but actively trigger systemic tolerance in *Cucurbita pepo*. Foliar applications effectively counteracted ZYMV-induced physiological damage by curbing lipid peroxidation, preserving photosynthetic integrity, and significantly upregulating the plant’s underlying enzymatic and non-enzymatic antioxidant networks. Importantly, this elicitor response was distinctly size- and dose-dependent, with the smallest nanocomposite formulation (CS-CuONPs, 17 nm) applied at 250 mg/L demonstrating the most robust disease suppression and vegetative growth recovery. Ultimately, this research provides a strong proof-of-concept that CS-CuO nanocomposites function as highly promising, eco-friendly therapeutic agents for managing ZYMV in sustainable agriculture. While our initial characterizations confirmed the critical dimensional and morphological parameters necessary for biological efficacy, we acknowledge the absence of Fourier-transform infrared spectroscopy (FTIR) and X-ray diffraction (XRD) data as a limitation of the current study. Future molecular profiling, comprehensive structural characterizations (including FTIR and XRD), and longitudinal field-scale studies will be vital to fully decipher the specific resistance pathways involved and to validate their long-term environmental safety.

## Supplementary Information

Supplementary Material 1.

## Data Availability

The datasets used and/or analyzed during the current investigation are available from the corresponding author on reasonable request.

## Abbreviations

ZYMV: Zucchini yellow mosaic virus
CS-CuO: Chitosan-loaded copper oxide nanocomposite
CSNPs: Chitosan nanoparticles
CuONPs: Copper oxide nanoparticles
NPs: Nanoparticles
RT-PCR: Reverse transcription polymerase chain reaction
P1: Protease 1 gene
TEM: Transmission electron microscopy
DLS: Dynamic light scattering
HC: Healthy control
IC: Infected control
DI%: Disease incidence percentage
DS%: Disease severity percentage
MDA: Malondialdehyde
ROS: Reactive oxygen species
APX: Ascorbate peroxidase
SOD: Superoxide dismutase
CAT: Catalase
GR: Glutathione reductase
PPO: Polyphenol oxidase
GSH: Reduced glutathione
Chl * a*: Chlorophyll *a*
Chl * b*: Chlorophyll *b*
FW: Fresh weight
DW: Dry weight
PCA: Principal component analysis
ARC: Agricultural Research Center
