# Supplementary figures and images for: Chitosan-loaded copper oxide nanocomposite as a promising antiviral alleviates Zucchini yellow mosaic virus infection in squash plants
Source: BMC Plant Biol. 2026 Jun 23;26:1075. doi: 10.1186/s12870-026-09268-1 (PMC13289537; doi:10.1186/s12870-026-09268-1)

**Original, full-length, and unprocessed gel image**

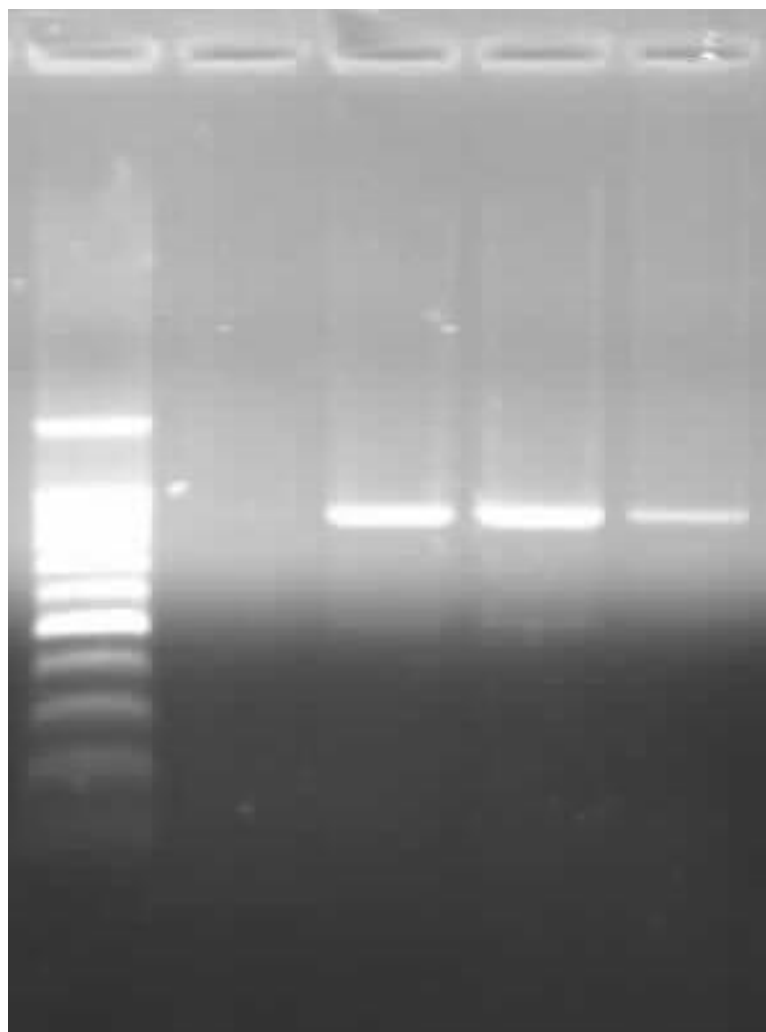

Supplement: Supplementary file 1 — Supplementary Material 1. [file 12870_2026_9268_MOESM1_ESM.pdf]
